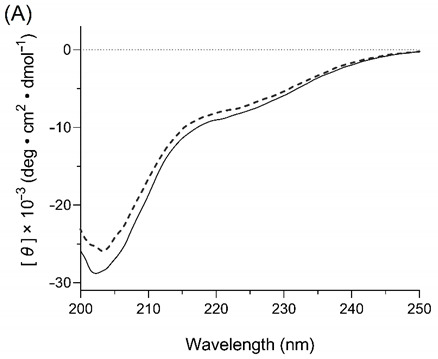# Correction: Sato et al. Design and Characterizations of Inhalable Poly(lactic-*co*-glycolic acid) Microspheres Prepared by the Fine Droplet Drying Process for a Sustained Effect of Salmon Calcitonin. *Molecules* 2020, *25*, 1311

**DOI:** 10.3390/molecules27206775

**Published:** 2022-10-11

**Authors:** Hideyuki Sato, Aiko Tabata, Tatsuru Moritani, Tadahiko Morinaga, Takahiro Mizumoto, Yoshiki Seto, Satomi Onoue

**Affiliations:** 1 Laboratory of Biopharmacy, School of Pharmaceutical Sciences, University of Shizuoka, 52-1 Yada, Suruga-ku, Shizuoka 422-8526, Japan; 2 Functional Material Development Center, Imaging Engine Development Division, RICOH Company, Ltd., 16-1 Honda-machi, Numazu-shi, Shizuoka 410-1458, Japan; 3 ILS Inc., 1-2-1 Kubogaoka, Moriya, Ibaraki 302-0104, Japan

The author wishes to make the following correction to this paper [1].

## Error in Figure

In the original publication, there was a mistake in Figure 2A as published. The published Figure 2A is a circular dichroism spectrum of salmon calcitonin samples. Since the title and unit of the y-axis and the range of x-axis were not appropriate for the data, a corrected version of Figure 2A has been attached. The authors apologize for any inconvenience caused and state that the scientific conclusions are unaffected. The original publication has also been updated.

Corrected Figure 2A: